# When Eating Disorders and Psychosis Collide: A Complex Clinical Case

**DOI:** 10.1192/j.eurpsy.2026.11231

**Published:** 2026-07-31

**Authors:** I. M. Peso Navarro, C. Garcia Cerdan, M. Ligero Argudo, I. Rodriguez Blanco, J. I. de la Iglesia Larrad, R. K. Gonzalez Bolaños

**Affiliations:** 1 Psiquiatría, Complejo Asistencial Universitario de Salamanca; 2 Psiquiatria, Complejo Asistencial Salamanca, Salamanca, Spain

## Abstract

**Introduction:**

Eating disorders (ED) and psychotic disorders are traditionally considered distinct clinical entities. However, their co-occurrence presents significant diagnostic and therapeutic challenges. Psychotic symptoms may emerge in the context of severe malnutrition, as part of a primary psychotic disorder, or as a comorbidity that worsens prognosis. Early identification and comprehensive management are essential to reduce morbidity and improve functional outcomes.

**Objectives:**

To describe a clinical case of co-occurring eating disorder and psychosis. To highlight the diagnostic challenges and differential considerations. To discuss therapeutic strategies and the importance of a multidisciplinary approach.

**Methods:**

We present a detailed clinical case of an adolescent patient with restrictive eating behaviors, rapid weight loss, and the subsequent emergence of psychotic symptoms. Information was obtained from clinical interviews, medical records, standardized psychometric assessments, and multidisciplinary team evaluations.

**Results:**

The patient initially presented with restrictive eating patterns, significant weight loss, and preoccupation with body image. During follow-up, psychotic features including paranoid delusions and auditory hallucinations emerged, complicating both diagnosis and treatment. Multiple interventions were implemented: nutritional rehabilitation, antipsychotic treatment, and family-based therapy. Partial remission of psychotic symptoms and gradual weight stabilization were achieved after several months of integrated care.

**Conclusions:**

This case illustrates the complex interplay between eating disorders and psychosis. The overlap of symptoms may delay diagnosis and hinder effective treatment. A comprehensive, multidisciplinary approach combining medical, psychiatric, and psychosocial interventions is crucial. Awareness of this comorbidity is necessary to optimize outcomes and guide future research.

**Disclosure of Interest:**

None Declared

